# Verification, validation, and uncertainty quantification of finite element analysis results for pedicle screw assemblies under ASTM F1717 flexion and extension testing

**DOI:** 10.3389/fbioe.2025.1623336

**Published:** 2025-12-08

**Authors:** On Sim, Byeong Cheol Jeong, Chiseung Lee

**Affiliations:** 1 Department of Biomedical Engineering, Graduate School, Pusan National University, Busan, Republic of Korea; 2 Department of Biomedical Engineering, School of Medicine, Pusan National University, Busan, Republic of Korea; 3 In Silico Medicine Lab, Biomedical Research Institute, Pusan National University Hospital, Busan, Republic of Korea

**Keywords:** ASTM standard F1717, finite element analysis (FEA), medical device digital tool (MDDT), ASME validation & verification (VandV) 40, pedicle screw, experiment, uncertainty quantification (UQ)

## Abstract

**Introduction:**

This study conducted verification, validation, and uncertainty quantification of finite element analysis (FEA) results for pedicle screw assemblies subjected to static flexion and extension loading conditions, in accordance with the ASTM F1717-15 standard.

**Methods:**

Four screw configurations and two material types, titanium alloy and titanium grade 23, were modeled to replicate the experimental setup. Simulations incorporated five types of contact conditions at the screw–block interface: frictionless; coefficient of friction (COF) of 0.1, 0.2, or 0.5; and bonded.

**Results:**

Experimental results demonstrated high repeatability, with maximum deviations of 6.4% for stiffness, 9.1% for yield displacement, 7.2% for yield force, and 7.5% for force at 20 mm displacement. The FEA results qualitatively captured the experimental trends but quantitatively overestimated mechanical responses, particularly under bonded contact conditions. The largest prediction errors were 19.8% for stiffness, 21.5% for yield force, and 18.4% for force at 20 mm, while the greatest deviation in yield displacement, 14.2%, occurred under frictionless conditions. When targeting an ASME VandV 40 Level 3 agreement (difference <10%), we found that no single interface model satisfied all validation metrics across screws and loading modes. When construct stiffness and force at 20 mm were prioritized as primary validation metrics, COF values in the range 0.10–0.20 yielded the most consistent agreement with experiment. Sensitivity analysis revealed that force and stiffness outputs were highly influenced by contact assumptions, whereas displacement outputs exhibited moderate sensitivity.

**Discussion:**

These findings highlight the importance of accurately defining contact conditions and experimentally characterizing interface behavior. To ensure predictive accuracy and regulatory relevance, validated friction models should be applied and contact parameters precisely calibrated, which can enhance the credibility of spinal biomechanics simulations. Relative to prior F1717-based simulations, this work quantifies the dominant impact of interface modeling and provides actionable parameter bounds for validation (μ = 0.10–0.20 for the tested constructs).

## 1 Introduction

Pedicle screws are widely used in spinal fusion, fracture stabilization, tumor resection, and corrective spinal procedures, serving as critical anchors to stabilize and realign the spine (Park et al., 2024; Jiang et al., 2025). Their mechanical performance is therefore essential for successful clinical outcomes.

In Korea, the Ministry of Food and Drug Safety (MFDS) requires mechanical performance testing for regulatory approval, typically following ASTM F1717 flexion, extension, rotation, and fatigue protocols (Baumann et al., 2021; Biswas et al., 2019; Rana et al., 2020; ASTM F1717 Standard, 2015, Ministry of Food and Drug Safety, 2025). The standard mandates five repetitions per mode, totaling 20 tests, each costing approximately USD 1,000 in materials alone, which represents a substantial burden for small manufacturers, particularly when retesting is required after a failure.

Computational Modeling and Simulation (CM&S) offers a promising route to reduce the time and cost of this process (Morrison et al., 2018). However, replacing physical tests with simulations requires demonstrated model credibility through rigorous Verification and Validation (VandV) and Uncertainty Quantification (UQ) (US Food and Drug Administration, 2016; US Food and Drug Administration, 2023). Relevant frameworks include ASME VandV 10 for computational solid mechanics and ASME VandV 40 for risk-informed assessment of medical device models (ASME V&V 10-2006, 2016; ASME V&V 40-2018, 2018).

Based on these standards, this study focuses on the uncertainty quantification necessary for applying computational models as a substitute for real mechanical testing. Following the ASME VandV 40–2018 guidelines, the study aims to identify and evaluate sources of uncertainty to ensure the credible use of computational modeling and simulation for pedicle screw performance evaluation.

## 2 Methods

### 2.1 Question of interest (?OI) and context of use (COU)

Question of Interest (?OI) as the specific question, decision, or concern that the computational model aims to address within its designated Context of Use (COU). The ?OI serves as the foundation for assessing model risk and establishing the credibility goals required for the model.

The COU defines the specific role and scope of the computational model used to address the ?OI. It should include a detailed statement of what will be modeled and how the outputs from the computational model will be used to answer or inform the ?OI.

The ?OI in this study is the feasibility of using computational modeling and simulation as a reliable alternative to ASTM F1717 experimental testing for pedicle screw regulatory approval. The context of use (COU) involves pedicle screws, which are primarily used in spinal fusion, fracture stabilization, tumor resection, and corrective surgeries. These screws function as anchors within the vertebrae to stabilize the spine. Given their critical role, the safety and performance of pedicle screws are essential considerations. Accordingly, for regulatory approval, their mechanical behavior is evaluated by assembling them into a construct and testing them according to ASTM F1717 guidelines.

### 2.2 Model risk

**FIGURE 1:**
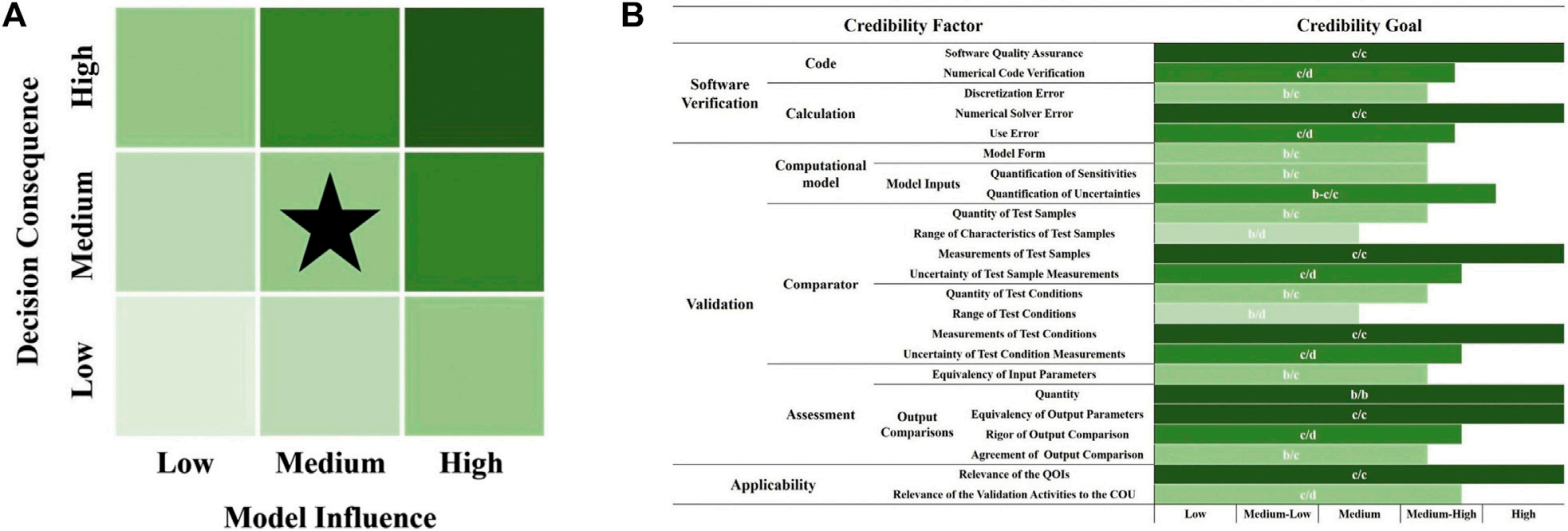
ASTM F1717 simulation **(A)** model risk; **(B)** Credibility goal.

Model risk is the possibility that the use of the computational model leads to a decision that results in patient harm and/or other undesirable impacts (Figure 1).

#### 2.2.1 Model influence

Model influence is the contribution of the computational model relative to other contributing evidence in making a decision. In this study, we set the model influence to medium because the simulation outputs from the computational model are a moderate factor in the decision (Smith et al., 2012; Dickman et al., 1992).

#### 2.2.2 Decision consequence

Decision consequence is the significance of an adverse outcome resulting from an incorrect decision. Consequences are typically considered in the context of potential harm to the patient (Stone, 2017; Bujold et al., 2022). In this study, we set the decision consequence to medium because an incorrect decision could result in minor patient injury or the need for physician intervention, or have other moderate impacts.

### 2.3 Model credibility

Model credibility refers to the level of trust in a computational model’s ability to accurately predict outcomes within its specified Context of Use (COU). It essentially determines whether the model is reliable enough to support decision-making processes, particularly in critical applications such as medical device development, where patient safety or performance outcomes are at stake (Figure 1) (Aldieri et al., 2023; Morrison et al., 2019).

Model credibility is typically established through verification and validation (VandV) activities. Verification ensures that the model’s implementation is correct, essentially confirming that the model was built correctly (Jeong et al., 2023). Validation determines whether the model accurately represents the real-world system it aims to simulate, ensuring that the right model was built (Courcelles et al., 2024).

### 2.4 Verification

#### 2.4.1 Code verification

Computational analyses were conducted using the commercially available finite element analysis (FEA) software platform, ANSYS Mechanical. The code verification activities were performed at levels that met the required model risk threshold, as the commercial FEA software employed established and documented software quality assurance procedures. Additionally, the release notes and anomaly lists associated with the software version ANSYS 2024 R2 were reviewed. No issues were identified in the software code that would affect the validity of this study.

#### 2.4.2 Calculation verification

Discretization error analysis was performed for the ASTM Standard F1717 pedicle screw assembly model. The pedicle screw assembly model consists of Ultra-High Molecular Weight Polyethylene (UHMWPE) blocks, pedicle screws, and spinal rods. Mesh refinement on the blocks, rods, and screws was performed to determine its impact on the maximum von Mises stress and reaction force in the assembly model. Construct stiffness was calculated as the slope of the elastic region, ranging from 2 mm to 10 mm displacement. Yield force was calculated using the 2% offset method, as specified in the ASTM Standard F1717. Additionally, the force at 20 mm displacement provided a post-yield quantity of interest (QOI).

### 2.5 Validation

#### 2.5.1 Computational model

**FIGURE 2:**
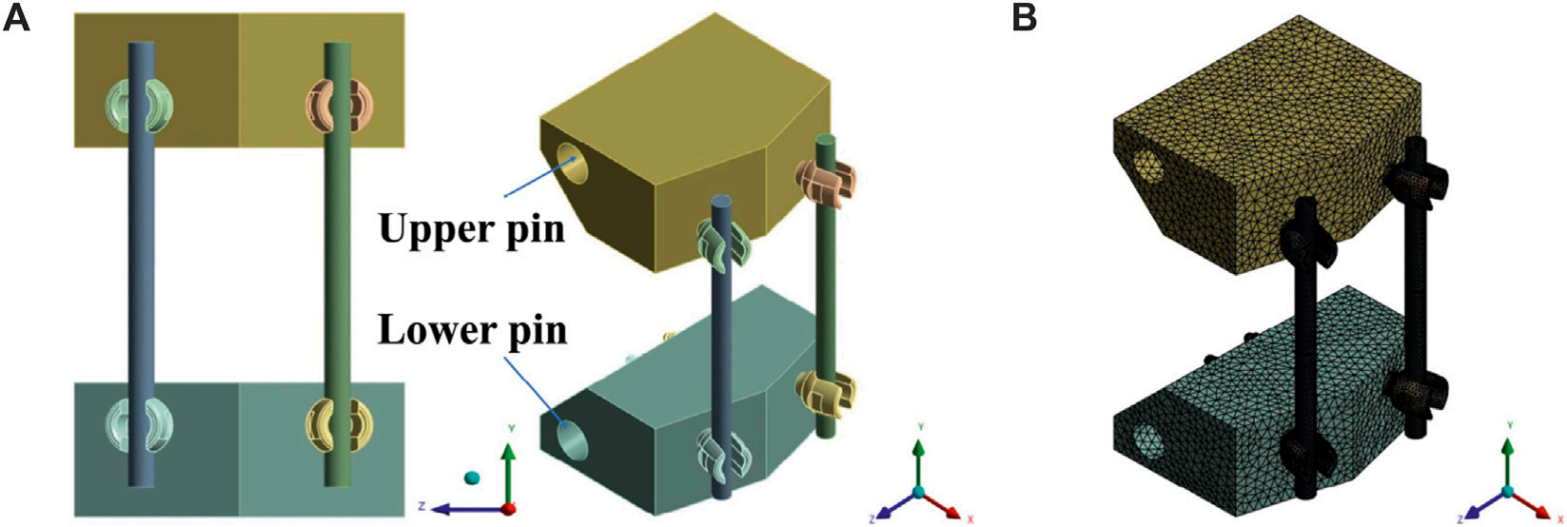
ASTM F1717 **(A)** 3D model; **(B)** Finite element model.

The computational model of the pedicle screw was reverse-engineered by acquiring a commercial pedicle screw and performing a 3D scan (Park et al., 2025). The UHMWPE block was modeled in 3D according to specifications, while the spinal rod was designed as a 3D model with a diameter of 6 mm and a length of 110 mm. And it was assembled in accordance with ASTM Standard F1717. The Finite Element (FE) model was constructed using the tetrahedral element C3D10 (Sim et al., 2022). This element type was selected to accurately represent the threads of the pedicle screw (Figure 2) (Kim et al., 2022).

##### 2.5.1.1 Model form

As shown in Figure 2, the pin and jig were not explicitly modeled. Instead, a remote point was assigned, and boundary conditions were directly applied to the surface of the block where the pin had been inserted. With the lower pin fixed, an axial displacement was applied to the upper pin to simulate static compression and tension tests. Each block was able to rotate around the pin axis.

##### 2.5.1.2 Model inputs

The UHMWPE blocks were modeled using a response function based on uniaxial tension test data from the ASTM Standard D638-14 test (ASTM D638-14 Standard, 2015). Spinal rods were simulated using a bilinear elastic-plastic material model based on the ASTM Standard E8 tensile test (ASTM E8 Standard, 2022). Pedicle screws were modeled with the same properties as spinal rods, as they must be manufactured from Ti-6Al-4V ELI (Titanium Grade 23) in accordance with approved material specifications (Table 1).

**TABLE 1:** Computational model material properties.

Material		Young’s modulus (GPa)	Poisson’s ratio
UHMWPE block		ASTM D638-14 test S-S curve	
Spinal rod (titanium grade 23)		ASTM E8 tensile test	
Pedicle screw	Titanium grade 23	108	0.34
	Titanium alloy	96	0.36

To further examine differences in material properties, we additionally incorporated the titanium alloy material properties provided by the ANSYS engineering database into the simulation. The simulation utilized implicit analysis, including the effects of large deformation.

To evaluate the influence of various model form assumptions on the mechanical performance of the construct, additional simulations were conducted. These simulations focused on key assumptions, including the boundary conditions at the screw-block interfaces and the material properties of the pedicle screw (Çetin and Bircan, 2021; Xu et al., 2019; Chen et al., 2003). At the screw-block interface, frictional contact with coefficients of 0.1, 0.2, and 0.5 was prescribed, informed by tribology reports for Ti–UHMWPE pairs (Leksycki et al., 2022) and recent F1717 modeling practice (Nagaraja et al., 2024). Bonded and frictionless contact were additionally analyzed as bounding cases to evaluate contact-assumption sensitivity.

###### 2.5.1.2.1 Quantification of sensitivities

A sensitivity analysis was performed to investigate the influence of variations in the coefficient of friction and Young’s modulus. Five different contact conditions were applied to two material types, namely, titanium alloy and titanium grade 23.

###### 2.5.1.2.2 Quantification of uncertainties

This component aims to investigate how uncertainties in the defined input parameters, specifically the coefficient of friction and Young’s modulus, are propagated through the simulation and influence the resulting outcomes.

#### 2.5.2 Comparator

The ASTM Standard F1717 static compression and tension tests were conducted using pedicle screws approved for surgical use in hospitals.

##### 2.5.2.1 Test samples

###### 2.5.2.1.1 Quantity of test samples

Each test was performed five times in accordance with ASTM Standard F1717 recommendations.

###### 2.5.2.1.2 Range of characteristics of test samples

The entire construct was assembled in accordance with ASTM Standard F1717 specifications. The spinal components were not intentionally fabricated at the extreme limits of specification tolerances; rather, they were assumed to fall within nominal dimensional ranges appropriate for standard testing protocols.

The spinal fusion construct consisted of a 6 mm diameter, 110 mm long medical-grade spinal rod (Ti-6Al-4V ELI), a UHMWPE block manufactured in compliance with ASTM Standard F1717, and mono-axial pedicle screws (Ti-6Al-4V ELI).

Four pedicle screw types (diameter × length) were evaluated: 6 × 40 mm, 6 × 50 mm, 7 × 40 mm, and 7 × 50 mm (Table 2).

**TABLE 2:** Properties of pedicle screw.

Screw type	Materials	Screw length (mm)	Thread length (mm)	Pitch (mm)	Thread diameter (mm)	Core diameter (mm)	Thread depth (mm)
640	Ti-6Al-4V ELI	53.58	40	2.45	6	4	1
650	Ti-6Al-4V ELI	63.58	50	2.45	6	4	1
740	Ti-6Al-4V ELI	53.55	40	2.45	7	5	1
750	Ti-6Al-4V ELI	63.69	50	2.45	7	5	1

###### 2.5.2.1.3 Measurements of test samples

Prior to testing, dimensional measurements were performed to ensure construct consistency (Kim et al., 2023). Specifically, the distance from the center of the upper pin axis to the center of the lower pin axis, as well as the distance between the upper and lower screws, was measured using a vernier caliper. To enhance measurement reliability, at least two independent observers performed the assessments.

###### 2.5.2.1.4 Uncertainty of test sample measurements

**FIGURE 3:**
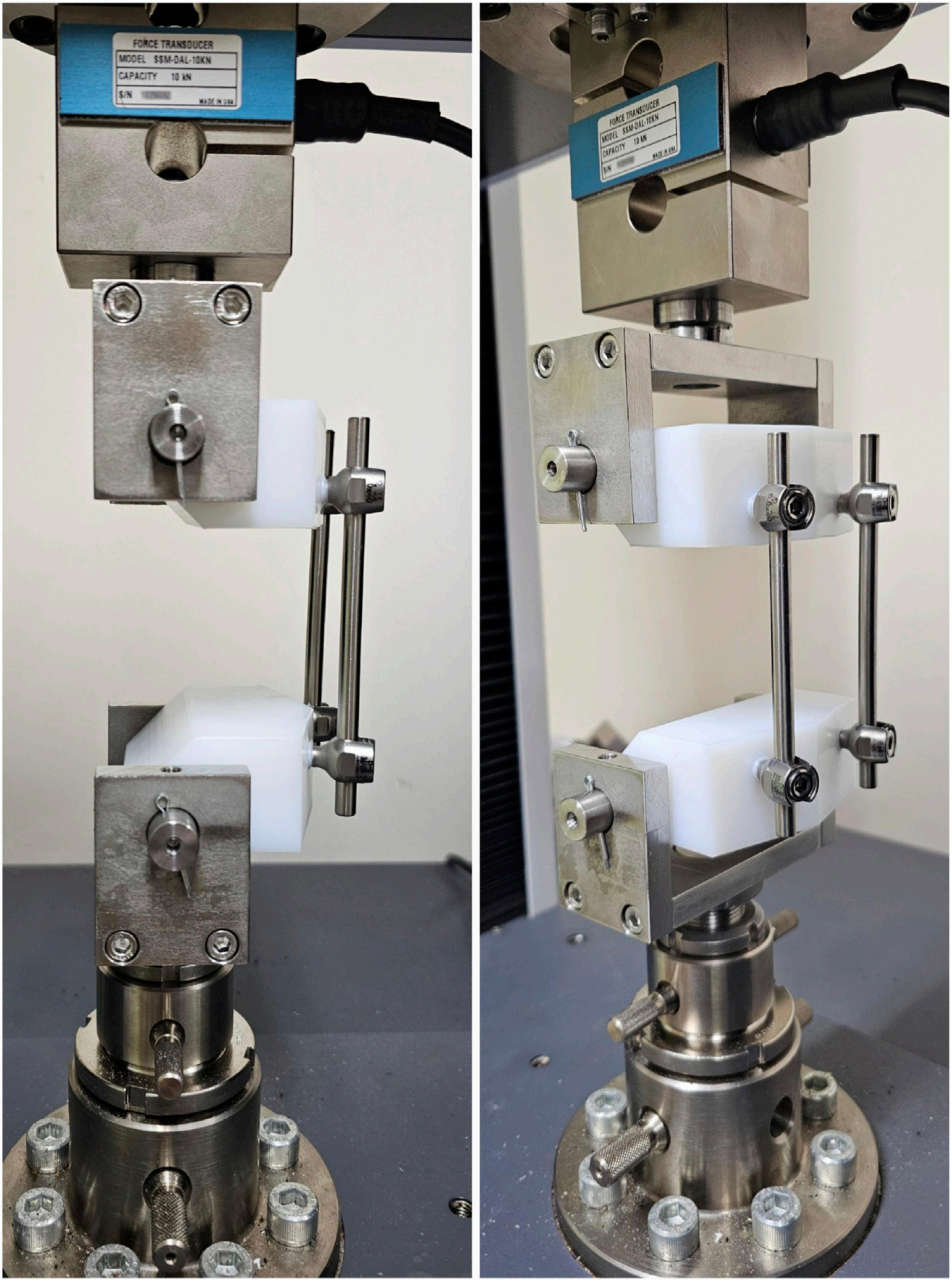
ASTM F1717 test image.

The single test method for static compression and tension was conducted using a calibrated universal testing machine (Shimadzu Corporation) at a speed of 25 mm/min, in accordance with ASTM Standard F1717-15, with a maximum displacement of 20 mm. Force–displacement data were recorded at 100 Hz (Figure 3).

##### 2.5.2.2 Test conditions

###### 2.5.2.2.1 Quantity of test conditions

In accordance with ASTM Standard F1717-15, the test was conducted under two test conditions (n = 2), compression and tension, at room temperature, using a loading speed of 25 mm/min and a maximum displacement of 20 mm.

###### 2.5.2.2.2 Range of test conditions

Two tests were performed within a range of conditions near the nominal values.

###### 2.5.2.2.3 Measurements of test conditions

This evaluation criterion assesses the extent to which test conditions were quantitatively and systematically measured. In this case, the test conditions, including temperature, loading speed, and displacement, were quantitatively defined and controlled.

###### 2.5.2.2.4 Uncertainty of test condition measurements

Environmental conditions were monitored and controlled with appropriate instrumentation: temperature was measured using a thermo-hygrometer, and loading speed and displacement were controlled using a universal testing machine that had been calibrated and verified by a qualified engineer. Consequently, a comprehensive quantification of uncertainty, encompassing factors such as repeatability, was achieved.

### 2.6 Verification activities

#### 2.6.1 Code verification

##### 2.6.1.1 Software quality assurance (SQA)

SQA procedures were clearly defined and systematically documented. The software anomaly list and development environment were thoroughly assessed to ensure complete understanding, and their impact on the COU was analyzed and recorded. Additionally, quality metrics were continuously monitored and tracked to uphold rigorous standards throughout the development process.

##### 2.6.1.2 Numerical code verification (NCV)

Benchmark verification problems were conducted to validate the proper functioning of software installations on their respective hardware systems. The obtained results demonstrated consistency with established benchmark problem solutions and exhibited convergence with mesh refinement, confirming the accuracy and reliability of the computational framework.

#### 2.6.2 Calculation verification

Calculation verification was performed through analyses of discretization error, numerical solver error (NSE), and use error.

##### 2.6.2.1 Discretization error

**FIGURE 4:**
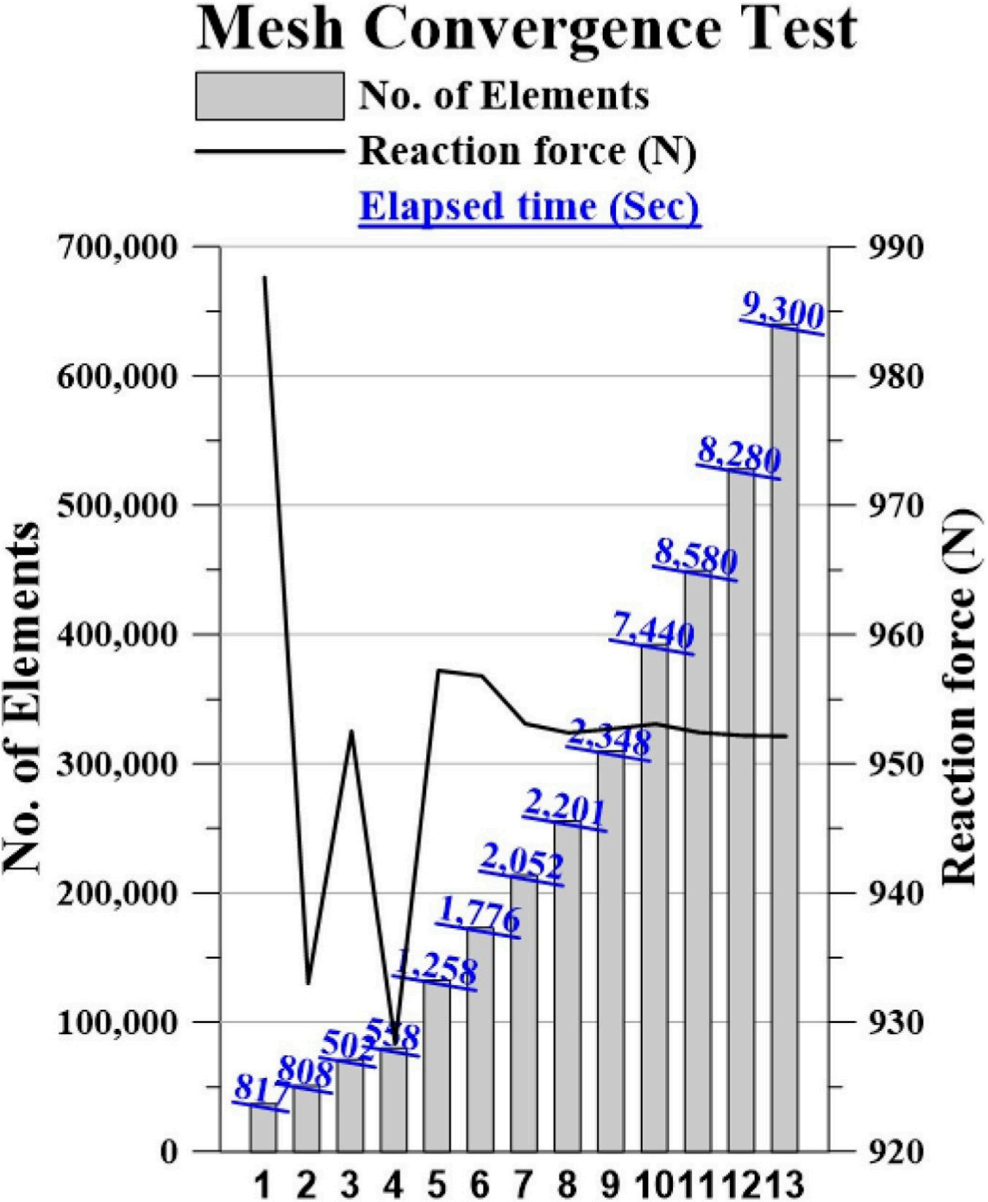
Mesh Convergence test graph.

To verify discretization error, the reaction force, which is identified as a quantity of interest (QOI) in the ASTM Standard F1717-15 assembly, has been observed to converge with a variation of less than 5% through mesh refinement applied to the UHMWPE block, spinal rod, and pedicle screw (Figure 4). In the final convergence step, the percent difference in reaction force between Case 8 and Case 9 was 0.04%, which is well below the target accuracy threshold of 5%. Therefore, Case 8 was selected for all subsequent simulations.

Convergence was achieved with an average element edge length of 3 mm for the UHMWPE block, 1 mm for the spinal rod, and 1 mm for the pedicle screw. Additionally, an element edge length of 1 mm was assigned separately for the contact surface between the UHMWPE block and the pedicle screw (Table 3).

**TABLE 3:** Mesh convergence test data.

Case	No. of elements	Reaction force (N)	Elapsed time (sec)
1	37,201	987.7	817
2	51,397	933.1	808
3	71,605	952.6	502
4	80,164	928.4	558
5	132,889	957.3	1,258
6	173,447	956.8	1,776
7	214,006	953.1	2,052
**8**	**256,087**	**952.4**	**2,201**
9	310,174	952.8	2,348
10	392,702	953.1	7,440
11	449,777	952.5	8,580
12	528,270	952.2	8,280
13	640,220	952.2	9,300

Bold values indicate the finite element model selected in this study.

##### 2.6.2.2 Numerical solver error (NSE)

Numerical solver error was quantified by varying the force and displacement convergence tolerances in the software. Simulations were conducted by increasing the convergence tolerances for force and displacement by factors of 2 and 4, respectively, relative to the default solver settings. The target numerical accuracy for the analysis was pre-specified as less than 5%, in line with commonly accepted standards for orthopedic finite element analyses.

##### 2.6.2.3 Use error

In order to identify potential use errors associated with the simulations, an internal peer review was conducted on the simulations. Key model inputs were verified, and no input errors were detected in any simulation.

### 2.7 Validation activities

**FIGURE 5:**
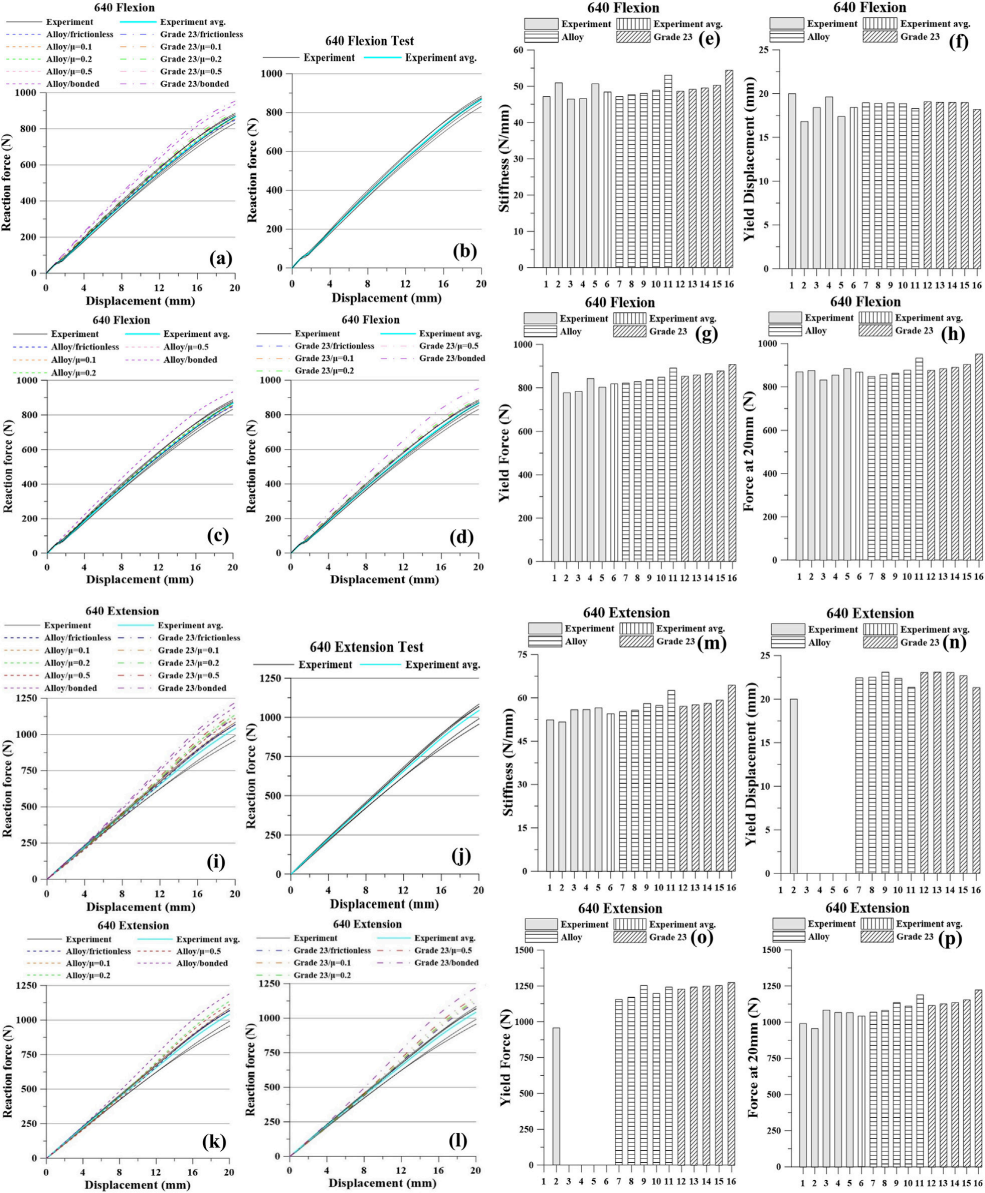
Pedicle screw 640 Graph: Flexion, **(a)** All Results; **(b)** Tests; **(c)** Titanium Alloy Simulations; **(d)** Titanium Grade 23 Simulations; **(e)** Stiffness; **(f)** Yield Displacement; **(g)** Yield Force; **(h)** Force at 20 mm; Extension, **(i)** All Results; **(j)** Tests; **(k)** Titanium Alloy Simulations; **(l)** Titanium Grade 23 Simulations; **(m)** Stiffness; **(n)** Yield Displacement; **(o)** Yield Force; **(p)** Force at 20 mm.

**FIGURE 6:**
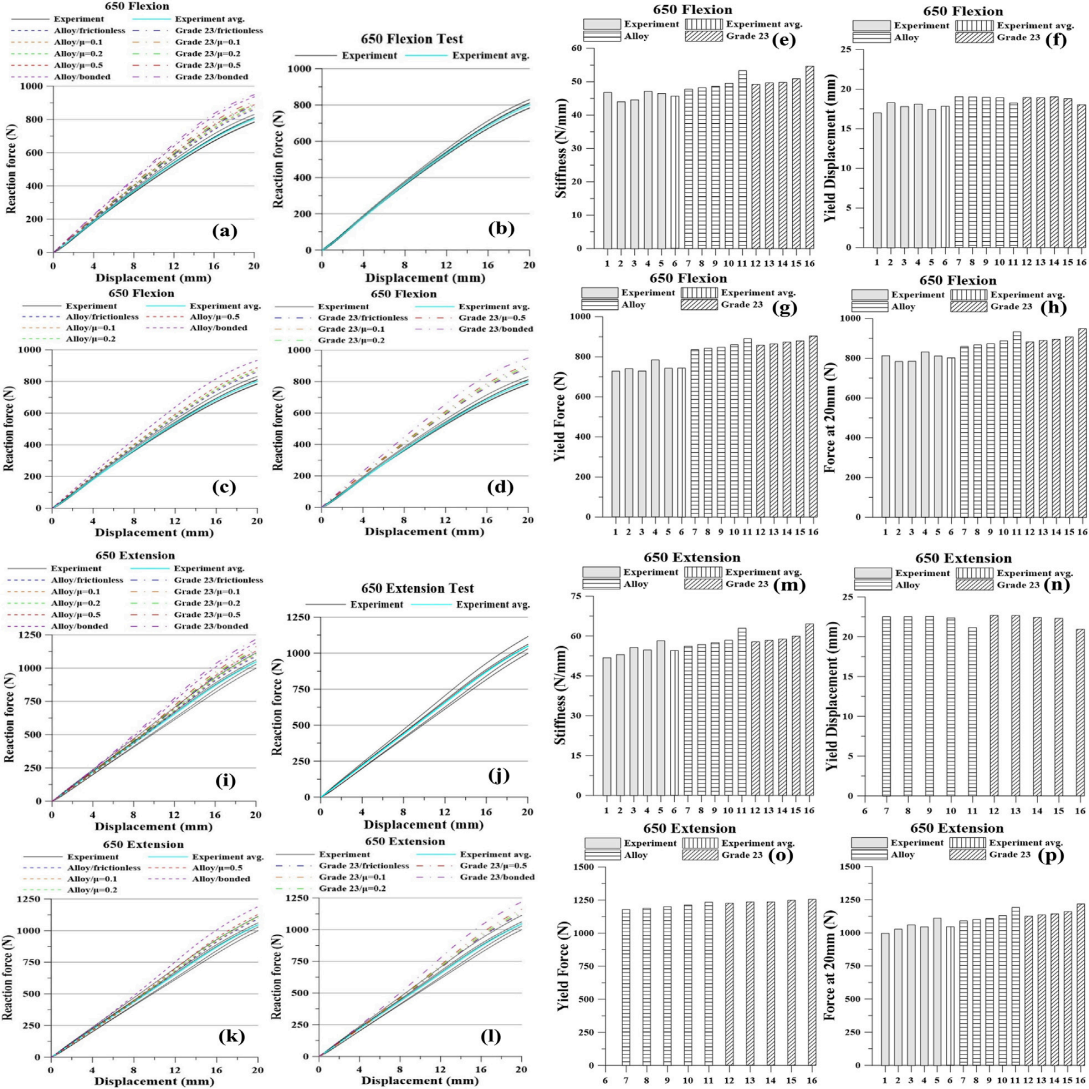
Pedicle screw 650 Graph: Flexion, **(a)** All Results; **(b)** Tests; **(c)** Titanium Alloy Simulations; **(d)** Titanium Grade 23 Simulations; **(e)** Stiffness; **(f)** Yield Displacement; **(g)** Yield Force; **(h)** Force at 20 mm; Extension, **(i)** All Results; **(j)** Tests; **(k)** Titanium Alloy Simulations; **(l)** Titanium Grade 23 Simulations; **(m)** Stiffness; **(n)** Yield Displacement; **(o)** Yield Force; **(p)** Force at 20 mm.

**FIGURE 7:**
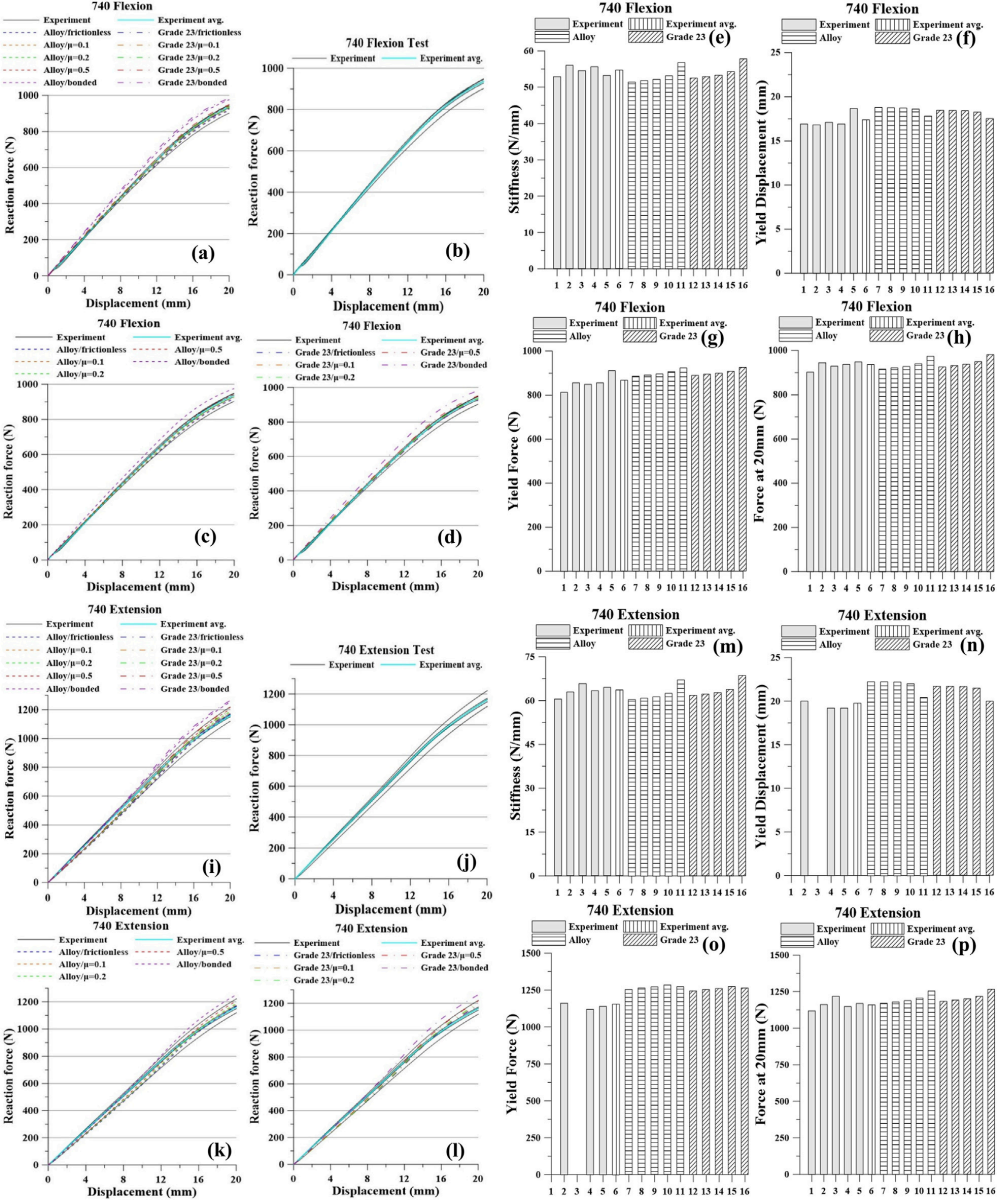
Pedicle screw 740 Graph: Flexion, **(a)** All Results; **(b)** Tests; **(c)** Titanium Alloy Simulations; **(d)** Titanium Grade 23 Simulations; **(e)** Stiffness; **(f)** Yield Displacement; **(g)** Yield Force; **(h)** Force at 20 mm; Extension, **(i)** All Results; **(j)** Tests; **(k)** Titanium Alloy Simulations; **(l)** Titanium Grade 23 Simulations; **(m)** Stiffness; **(n)** Yield Displacement; **(o)** Yield Force; **(p)** Force at 20 mm.

**FIGURE 8:**
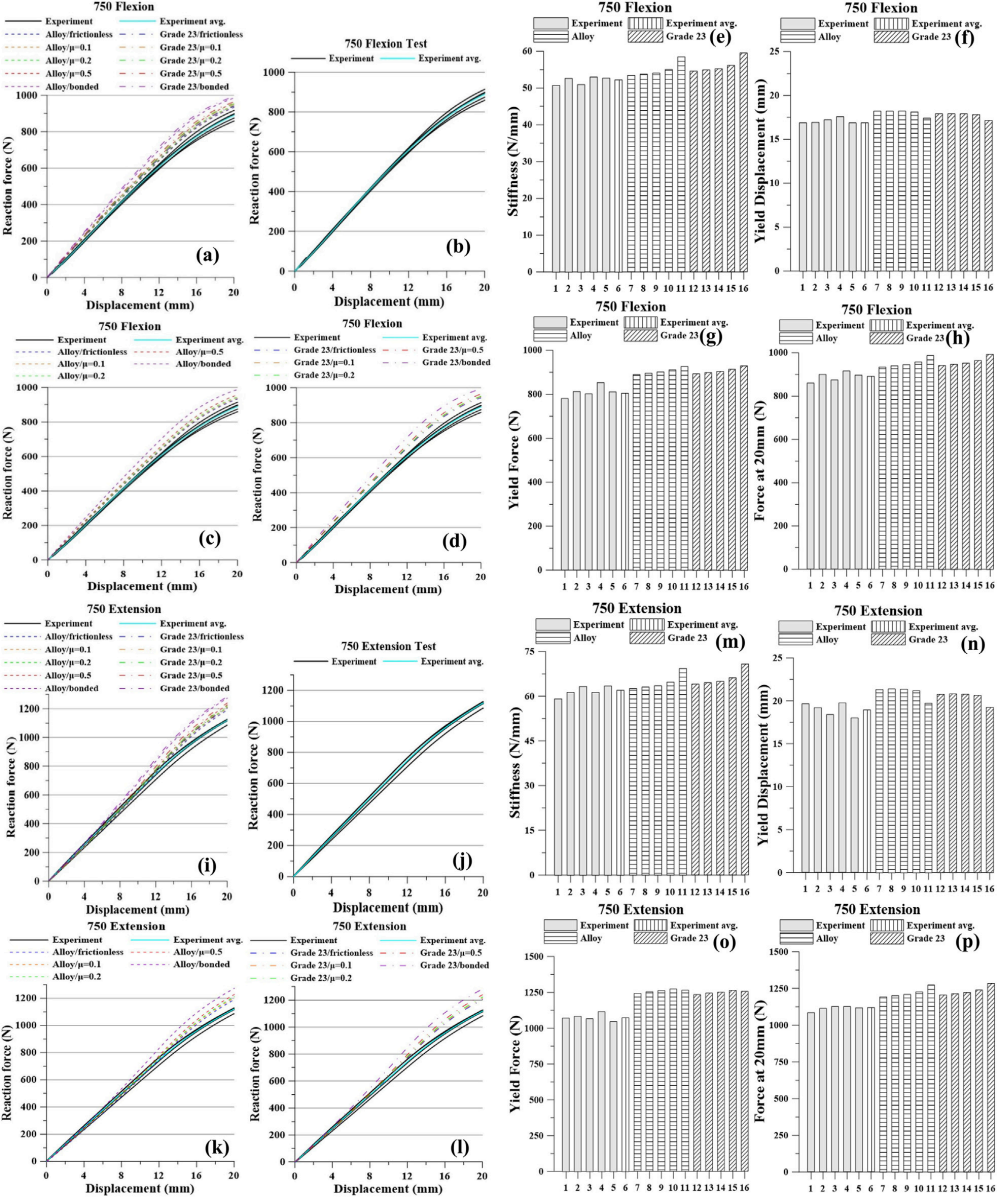
Pedicle screw 750 Graph: Flexion, **(a)** All Results; **(b)** Tests; **(c)** Titanium Alloy Simulations; **(d)** Titanium Grade 23 Simulations; **(e)** Stiffness; **(f)** Yield Displacement; **(g)** Yield Force; **(h)** Force at 20 mm; Extension, **(i)** All Results; **(j)** Tests; **(k)** Titanium Alloy Simulations; **(l)** Titanium Grade 23 Simulations; **(m)** Stiffness; **(n)** Yield Displacement; **(o)** Yield Force; **(p)** Force at 20 mm.

The experimental and simulation results are presented in Figures 5–8 and Tables 4–7. For each screw type (640, 650, 740, and 750), five flexion tests and five extension tests were performed. Values in brackets indicate the percent (%) difference relative to the average, computed from all measurements.

**TABLE 4:** ASTM standard F1717-15 Test&Simulation-640.

Pedicle screw 640 flexion test					
[%]Difference relative to the average	Case	Stiffness (N/mm)	Yield displacement (mm)	Yield force (N)	Force at 20 mm (N)
Experiment	1. Test-1	47.14 [-2.5]	19.97 [9.1]	870.00 [7.2]	870.03 [0.7]
	2. Test-2	50.92 [5.3]	16.79 [-8.3]	777.60 [-4.2]	875.95 [1.4]
	3. Test-3	46.45 [-3.9]	18.38 [0.4]	783.60 [-3.5]	832.75 [-3.6]
	4. Test-4	46.60 [-3.6]	19.60 [7.1]	843.00 [3.9]	854.69 [-1.1]
	5. Test-5	50.67 [4.8]	17.36 [-5.2]	802.80 [-1.1]	884.84 [2.4]
	6. Average	48.36 [0.0]	18.30 [0.0]	811.67 [0.0]	864.08 [0.0]
Titanium alloy	7. Frictionless	47.13 [-2.5]	18.95 [3.5]	822.00 [1.3]	848.58 [-1.8]
	8. COF 0.1	47.64 [-1.5]	18.90 [3.3]	828.40 [2.1]	856.61 [-0.9]
	9. COF 0.2	48.01 [-0.7]	18.94 [3.5]	836.80 [3.1]	863.82 [0.0]
	10. COF 0.5	48.89 [1.1]	18.87 [3.1]	848.60 [4.6]	877.94 [1.6]
	11. Bonded	53.00 [9.6]	18.32 [0.1]	891.00 [9.8]	933.66 [8.1]
Titanium grade 23	12. Frictionless	48.60 [0.5]	19.06 [4.1]	853.00 [5.1]	877.07 [1.5]
	13. COF 0.1	49.11 [1.6]	19.01 [3.9]	859.00 [5.8]	884.52 [2.4]
	14. COF 0.2	49.49 [2.3]	18.99 [3.8]	865.00 [6.6]	891.01 [3.1]
	15. COF 0.5	50.25 [3.9]	18.98 [3.7]	877.60 [8.1]	904.21 [4.6]
	16. Bonded	54.40 [12.5]	18.19 [-0.6]	907.20 [11.8]	952.44 [10.2]
Pedicle screw 640 extension test					
Experiment	1. Test-1	52.45 [-3.8]	-	-	990.97 [-4.1]
	2. Test-2	51.73 [-5.2]	20.00	955.88	955.88 [-7.5]
	3. Test-3	55.95 [2.6]	-	-	1,083.86 [4.9]
	4. Test-4	56.02 [2.7]	-	-	1,067.61 [3.3]
	5. Test-5	56.58 [3.7]	-	-	1,066.31 [3.2]
	6. Average	54.55 [0.0]	-	-	1,033.14 [0.0]
Titanium alloy	7. Frictionless	55.21 [1.2]	22.41	1,154.67	1,070.00 [3.6]
	8. COF 0.1	55.80 [2.3]	22.49	1,170.33	1,081.80 [4.7]
	9. COF 0.2	58.04 [6.4]	23.07	1,251.33	1,135.90 [9.9]
	10. COF 0.5	57.43 [5.3]	22.35	1,196.33	1,111.50 [7.6]
	11. Bonded	62.55 [14.7]	21.32	1,240.00	1,189.40 [15.1]
Titanium grade 23	12. Frictionless	57.05 [4.6]	23.04	1,229.33	1,116.60 [8.1]
	13. COF 0.1	57.63 [5.7]	23.08	1,242.67	1,128.10 [9.2]
	14. COF 0.2	58.12 [6.5]	23.05	1,251.00	1,136.10 [10.0]
	15. COF 0.5	59.25 [8.6]	22.67	1,253.67	1,155.50 [11.8]
	16. Bonded	64.35 [18.0]	21.30	1,274.67	1,223.00 [18.4]

**TABLE 5:** ASTM standard F1717-15 Test&Simulation-650.

Pedicle screw 650 flexion test					
[%]Difference relative to the average	Case	Stiffness (N/mm)	Yield displacement (mm)	Yield force (N)	Force at 20 mm (N)
Experiment	1. Test-1	46.86 [2.3]	17.03 [-4.2]	727.20 [-2.3]	812.19 [1.0]
	2. Test-2	44.05 [-3.9]	18.30 [3.0]	739.40 [-0.7]	782.97 [-2.7]
	3. Test-3	44.60 [-2.7]	17.83 [0.3]	727.60 [-2.3]	784.46 [-2.5]
	4. Test-4	47.11 [2.8]	18.14 [2.1]	783.20 [5.2]	831.63 [3.4]
	5. Test-5	46.51 [1.5]	17.46 [-1.7]	741.60 [-0.4]	811.04 [0.8]
	6. Average	45.83 [0.0]	17.77 [0.0]	744.60 [0.0]	804.46 [0.0]
Titanium alloy	7. Frictionless	47.73 [4.2]	19.03 [7.1]	836.20 [12.3]	860.44 [7.0]
	8. COF 0.1	48.20 [5.2]	19.01 [7.0]	843.40 [13.3]	868.23 [7.9]
	9. COF 0.2	48.62 [6.1]	18.96 [6.7]	848.00 [13.9]	874.53 [8.7]
	10. COF 0.5	49.50 [8.0]	18.91 [6.4]	861.00 [15.6]	888.53 [10.5]
	11. Bonded	53.28 [16.3]	18.25 [2.7]	891.60 [19.7]	934.78 [16.2]
Titanium grade 23	12. Frictionless	49.20 [7.4]	18.95 [6.6]	857.80 [15.2]	883.98 [9.9]
	13. COF 0.1	49.64 [8.3]	18.92 [6.5]	864.20 [16.1]	891.08 [10.8]
	14. COF 0.2	49.81 [8.7]	19.04 [7.1]	873.20 [17.3]	896.98 [11.5]
	15. COF 0.5	50.88 [11.0]	18.79 [5.7]	879.20 [18.1]	909.44 [13.1]
	16. Bonded	54.66 [19.3]	18.04 [1.5]	903.40 [21.3]	950.36 [18.1]
Pedicle screw 650 extension test					
Experiment	1. Test-1	51.84 [-5.2]	-	-	997.62 [-4.9]
	2. Test-2	53.03 [-3.1]	-	-	1,028.82 [-2.0]
	3. Test-3	55.67 [1.8]	-	-	1,061.17 [1.1]
	4. Test-4	54.77 [0.1]	-	-	1,046.95 [-0.2]
	5. Test-5	58.20 [6.4]	-	-	1,112.63 [6.0]
	6. Average	54.70 [0.0]	-	-	1,049.55 [0.0]
Titanium alloy	7. Frictionless	56.05 [2.5]	22.53	1,179.00	1,090.10 [3.9]
	8. COF 0.1	56.53 [3.3]	22.54	1,188.00	1,100.30 [4.8]
	9. COF 0.2	57.33 [4.8]	22.57	1,200.33	1,109.00 [5.7]
	10. COF 0.5	58.18 [6.4]	22.38	1,213.67	1,129.20 [7.6]
	11. Bonded	62.82 [14.8]	21.15	1,234.67	1,191.20 [13.5]
Titanium grade 23	12. Frictionless	57.86 [5.8]	22.68	1,225.67	1,127.20 [7.4]
	13. COF 0.1	58.36 [6.7]	22.68	1,234.67	1,136.80 [8.3]
	14. COF 0.2	59.05 [7.9]	22.44	1,235.33	1,144.60 [9.1]
	15. COF 0.5	59.95 [9.6]	22.30	1,247.00	1,161.60 [10.7]
	16. Bonded	64.58 [18.1]	20.93	1,254.67	1,218.80 [16.1]

**TABLE 6:** ASTM standard F1717-15 Test&Simulation-740.

Pedicle screw 740 flexion test					
[%]Difference relative to the average	Case	Stiffness (N/mm)	Yield displacement (mm)	Yield force (N)	Force at 20 mm (N)
Experiment	1. Test-1	52.87 [-2.9]	16.91 [-2.0]	814.00 [-5.0]	902.14 [-3.3]
	2. Test-2	56.01 [2.9]	16.82 [-2.5]	857.20 [0.0]	945.14 [1.3]
	3. Test-3	54.54 [0.2]	17.10 [-0.9]	850.00 [-0.8]	929.84 [-0.3]
	4. Test-4	55.62 [2.1]	16.92 [-2.0]	857.00 [0.0]	936.99 [0.5]
	5. Test-5	53.22 [-2.3]	18.65 [8.1]	912.20 [6.4]	948.98 [1.8]
	6. Average	54.45 [0.0]	17.26 [0.0]	857.00 [0.0]	932.62 [0.0]
Titanium alloy	7. Frictionless	51.41 [-5.6]	18.75 [8.7]	886.20 [3.4]	916.79 [-1.7]
	8. COF 0.1	51.80 [-4.9]	18.72 [8.5]	891.20 [4.0]	922.79 [-1.1]
	9. COF 0.2	52.18 [-4.2]	18.69 [8.3]	896.00 [4.6]	928.43 [-0.4]
	10. COF 0.5	53.11 [-2.5]	18.57 [7.6]	906.20 [5.7]	941.06 [0.9]
	11. Bonded	56.75 [4.2]	17.78 [3.0]	923.20 [7.7]	975.16 [4.6]
Titanium grade 23	12. Frictionless	52.52 [-3.5]	18.44 [6.9]	888.80 [3.7]	926.36 [-0.7]
	13. COF 0.1	52.92 [-2.8]	18.41 [6.7]	894.40 [4.4]	932.64 [0.0]
	14. COF 0.2	53.30 [-2.1]	18.38 [6.5]	898.80 [4.9]	938.14 [0.6]
	15. COF 0.5	54.30 [-0.3]	18.23 [5.6]	907.80 [5.9]	950.41 [1.9]
	16. Bonded	57.79 [6.1]	17.52 [1.5]	925.20 [8.0]	982.32 [5.3]
Pedicle screw 740 extension test					
Experiment	1. Test-1	60.43 [-4.7]	-	-	1,118.33 [-3.9]
	2. Test-2	62.92 [-0.7]	20.00 [1.3]	1,161.66 [0.5]	1,161.66 [-0.1]
	3. Test-3	65.76 [3.7]	-	-	1,218.09 [4.7]
	4. Test-4	63.34 [-0.1]	19.20 [-2.8]	1,120.23 [-3.0]	1,147.76 [-1.3]
	5. Test-5	64.47 [1.7]	19.20 [-2.8]	1,140.30 [-1.3]	1,168.86 [0.5]
	6. Average	63.39 [0.0]	19.75 [0.0]	1,155.33 [0.0]	1,163.14 [0.0]
Titanium alloy	7. Frictionless	60.42 [-4.7]	22.24 [12.6]	1,252.00 [8.4]	1,170.00 [0.6]
	8. COF 0.1	60.90 [-3.9]	22.24 [12.6]	1,262.00 [9.2]	1,179.10 [1.4]
	9. COF 0.2	61.37 [-3.2]	22.22 [12.5]	1,270.00 [9.9]	1,187.60 [2.1]
	10. COF 0.5	62.54 [-1.3]	22.01 [11.5]	1,282.00 [11.0]	1,205.70 [3.7]
	11. Bonded	67.17 [6.0]	20.44 [3.5]	1,271.00 [10.0]	1,254.20 [7.8]
Titanium grade 23	12. Frictionless	61.75 [-2.6]	21.66 [9.7]	1,243.33 [7.6]	1,183.10 [1.7]
	13. COF 0.1	62.22 [-1.8]	21.66 [9.7]	1,253.00 [8.5]	1,192.00 [2.5]
	14. COF 0.2	62.67 [-1.1]	21.63 [9.5]	1,260.33 [9.1]	1,200.20 [3.2]
	15. COF 0.5	63.80 [0.7]	21.47 [8.7]	1,273.00 [10.2]	1,217.80 [4.7]
	16. Bonded	68.53 [8.1]	19.95 [1.0]	1,263.50 [9.4]	1,265.10 [8.8]

**TABLE 7:** ASTM standard F1717-15 Test&Simulation-750.

Pedicle screw 750 flexion test					
[%]Difference relative to the average	Case	Stiffness (N/mm)	Yield displacement (mm)	Yield force (N)	Force at 20 mm (N)
Experiment	1. Test-1	50.75 [-2.5]	16.89 [-1.3]	780.20 [-3.8]	859.25 [-3.3]
	2. Test-2	52.66 [1.2]	16.93 [-1.1]	811.80 [0.1]	899.02 [1.2]
	3. Test-3	50.97 [-2.0]	17.24 [0.8]	801.60 [-1.2]	873.89 [-1.7]
	4. Test-4	53.02 [1.9]	17.59 [2.8]	852.00 [5.0]	915.31 [3.0]
	5. Test-5	52.73 [1.4]	16.88 [-1.3]	810.20 [-0.1]	896.15 [0.8]
	6. Average	52.03 [0.0]	17.11 [0.0]	811.20 [0.0]	888.73 [0.0]
Titanium alloy	7. Frictionless	53.39 [2.6]	18.20 [6.4]	890.80 [9.8]	933.23 [5.0]
	8. COF 0.1	53.73 [3.3]	18.20 [6.4]	896.80 [10.6]	939.22 [5.7]
	9. COF 0.2	54.05 [3.9]	18.21 [6.4]	902.60 [11.3]	944.62 [6.3]
	10. COF 0.5	54.98 [5.7]	18.11 [5.8]	912.40 [12.5]	957.04 [7.7]
	11. Bonded	58.40 [12.3]	17.40 [1.7]	927.40 [14.3]	986.73 [11.0]
Titanium grade 23	12. Frictionless	54.56 [4.9]	17.90 [4.6]	894.00 [10.2]	942.09 [6.0]
	13. COF 0.1	54.90 [5.5]	17.90 [4.6]	899.60 [10.9]	947.77 [6.6]
	14. COF 0.2	55.21 [6.1]	17.90 [4.6]	905.00 [11.6]	952.91 [7.2]
	15. COF 0.5	56.10 [7.8]	17.81 [4.1]	914.40 [12.7]	964.85 [8.6]
	16. Bonded	59.48 [14.3]	17.15 [0.2]	930.00 [14.6]	993.24 [11.8]
Pedicle screw 750 extension test					
Experiment	1. Test-1	59.16 [-4.2]	19.63 [3.7]	1,071.93 [-0.3]	1,083.88 [-2.6]
	2. Test-2	61.36 [-0.6]	19.18 [1.3]	1,083.83 [0.8]	1,112.29 [0.0]
	3. Test-3	63.32 [2.6]	18.37 [-3.0]	1,067.50 [-0.7]	1,125.01 [1.1]
	4. Test-4	61.32 [-0.7]	19.73 [4.2]	1,116.97 [3.9]	1,125.12 [1.1]
	5. Test-5	63.51 [2.9]	18.00 [-4.9]	1,047.20 [-2.6]	1,116.69 [0.4]
	6. Average	61.73 [0.0]	18.93 [0.0]	1,074.70 [0.0]	1,112.73 [0.0]
Titanium alloy	7. Frictionless	62.60 [1.4]	21.34 [12.7]	1,241.17 [15.5]	1,193.10 [7.2]
	8. COF 0.1	62.99 [2.0]	21.42 [13.1]	1,253.27 [16.6]	1,201.10 [7.9]
	9. COF 0.2	63.46 [2.8]	21.39 [13.0]	1,260.60 [17.3]	1,209.20 [8.7]
	10. COF 0.5	64.61 [4.7]	21.21 [12.0]	1,271.97 [18.4]	1,226.60 [10.2]
	11. Bonded	69.32 [12.3]	19.75 [4.3]	1,263.73 [17.6]	1,273.50 [14.4]
Titanium grade 23	12. Frictionless	64.03 [3.7]	20.77 [9.7]	1,232.37 [14.7]	1,205.30 [8.3]
	13. COF 0.1	64.38 [4.3]	20.83 [10.0]	1,243.00 [15.7]	1,212.80 [9.0]
	14. COF 0.2	64.84 [5.0]	20.80 [9.9]	1,249.60 [16.3]	1,220.50 [9.7]
	15. COF 0.5	65.99 [6.9]	20.64 [9.0]	1,261.33 [17.4]	1,237.80 [11.2]
	16. Bonded	70.73 [14.6]	19.26 [1.7]	1,255.33 [16.8]	1,283.60 [15.4]

#### 2.7.1 Model form analysis

Sensitivity analysis was conducted to investigate the contact and interaction conditions between the UHMWPE block and the pedicle screw. In this study, the thread geometry of the pedicle screw was accurately modeled in three dimensions using 3D scanning equipment to replicate the actual screw shape. Two different material types, titanium alloy and titanium grade 23, were used to define the mechanical properties of the pedicle screw.

Five contact conditions between the UHMWPE block and the pedicle screw were considered: frictionless, coefficient of friction (COF) values of 0.1, 0.2, 0.5, and bonded. Additionally, the interface between the pedicle screw and spinal rod was modeled as bonded.

The experimental and simulation results indicated that the frictionless contact condition between the UHMWPE block and the pedicle screw had the most significant impact, resulting in a maximum stiffness reduction of 11.7% compared to the bonded condition. In terms of yield displacement, the frictionless condition showed a maximum increase of 11.5%. Yield force exhibited a maximum reduction of 7.7%, and the force at 20 mm decreased by up to 10% under the frictionless condition relative to the bonded condition.

#### 2.7.2 Sensitivity analysis

The sensitivity analysis results were obtained by applying five different contact conditions to two material types (titanium alloy and titanium grade 23). The variable having the greatest impact on stiffness was the contact condition, showing a maximum difference of 19.8% compared to the experimental mean value. The contact condition also had the most significant effect on yield displacement, with a maximum difference of 14.2%. Similarly, yield force showed a maximum variation of 21.5%, and the force at 20 mm exhibited a maximum difference of 18.4%, both due to variations in the contact condition.

## 3 Results

### 3.1 Static compression and tension data

#### 3.1.1 Pedicle screw 640 compression and tension

Under the 640 flexion condition, the experimental averages were: stiffness 48.36 N/mm, yield displacement 18.30 mm, yield force 811.67 N, and force at 20 mm 864.08 N. As contact constraint increased from frictionless to frictional to bonded, the model predicted higher stiffness, yield force, and force at 20 mm, with a slight decrease in yield displacement. For the Ti alloy model, stiffness increased from 47.13 to 53.00 N/mm, yield force from 822.00 to 891.00 N, and the force at 20 mm from 848.58 to 933.66 N, while yield displacement decreased from 18.95 to 18.32 mm. For Ti Grade 23, stiffness rose from 48.60 to 54.40 N/mm, yield force from 853.00 to 907.20 N, and the force at 20 mm from 877.07 to 952.44 N, while yield displacement decreased from 19.06 to 18.19 mm.

Under 640 extension, experiment averaged stiffness 54.55 N/mm and force at 20 mm 1,033.14 N (one specimen: 20.00 mm yield displacement, 955.88 N yield force). In simulation, tightening the contact from frictionless → frictional → bonded increased stiffness and the force at 20 mm and slightly reduced yield displacement. Ti alloy: stiffness 55.21→62.55 N/mm, yield displacement 22.41→21.32 mm, yield force 1,154.67→1,240.00 N, force at 20 mm 1,070.00→1,189.40 N. Ti Grade 23: stiffness 57.05→64.35 N/mm, yield displacement 23.04→21.30 mm, yield force 1,229.33→1,274.67 N, force at 20 mm 1,116.60→1,223.00 N.

#### 3.1.2 Pedicle screw 650 compression and tension

Under the 650 flexion condition, the experimental averages were: stiffness 45.83 N/mm, yield displacement 17.77 mm, yield force 744.60 N, and force at 20 mm 804.46 N. As contact constraint increased from frictionless to frictional to bonded, the model predicted higher stiffness, yield force, and force at 20 mm, with a slight decrease in yield displacement. For the Ti alloy model, stiffness increased from 47.73 to 53.28 N/mm, yield force from 836.20 to 891.60 N, and the force at 20 mm from 860.44 to 934.78 N, while yield displacement decreased from 19.03 to 18.25 mm. For Ti Grade 23, stiffness rose from 49.20 to 54.66 N/mm, yield force from 857.80 to 903.40 N, and the force at 20 mm from 883.98 to 950.36 N, while yield displacement decreased from 18.95 to 18.04 mm.

Under 650 extension, experiment averaged stiffness 54.70 N/mm and force at 20 mm 1,049.55 N. In simulation, tightening the contact from frictionless → frictional → bonded increased stiffness and the force at 20 mm and slightly reduced yield displacement. Ti alloy: stiffness 56.05→62.82 N/mm, yield displacement 22.53→21.15 mm, yield force 1,179.00→1,234.67 N, force at 20 mm 1,090.10→1,191.20 N. Ti Grade 23: stiffness 57.05→64.35 N/mm, yield displacement 22.68→20.93 mm, yield force 1,225.67→1,254.67 N, force at 20 mm 1,127.20→1,218.80 N.

#### 3.1.3 Pedicle screw 740 compression and tension

Under the 740 flexion condition, the experimental averages were: stiffness 54.45 N/mm, yield displacement 17.26 mm, yield force 857.00 N, and force at 20 mm 932.62 N. As contact constraint increased from frictionless to frictional to bonded, the model predicted higher stiffness, yield force, and force at 20 mm, with a slight decrease in yield displacement. For the Ti alloy model, stiffness increased from 51.41 to 56.75 N/mm, yield force from 886.20 to 923.20 N, and the force at 20 mm from 916.79 to 975.16 N, while yield displacement decreased from 18.75 to 17.78 mm. For Ti Grade 23, stiffness rose from 52.52 to 57.79 N/mm, yield force from 888.80 to 925.20 N, and the force at 20 mm from 926.36 to 982.32 N, while yield displacement decreased from 18.44 to 17.52 mm.

Under 740 extension, experiment averaged stiffness 63.39 N/mm, yield displacement 19.75 mm, yield force 1,155.33 N, and force at 20 mm 1,163.14 N. In simulation, tightening the contact from frictionless → frictional → bonded increased stiffness and the force at 20 mm and slightly reduced yield displacement. Ti alloy: stiffness 60.42→67.17 N/mm, yield displacement 22.24→20.44 mm, yield force 1,252.00→1,271.00 N, force at 20 mm 1,170.00→1,254.20 N. Ti Grade 23: stiffness 61.75→68.53 N/mm, yield displacement 21.66→19.95 mm, yield force 1,243.33→1,263.50 N, force at 20 mm 1,183.10→1,265.10 N.

#### 3.1.4 Pedicle screw 750 compression and tension

Under the 750 flexion condition, the experimental averages were: stiffness 52.03 N/mm, yield displacement 17.11 mm, yield force 811.20 N, and force at 20 mm 888.73 N. As contact constraint increased from frictionless to frictional to bonded, the model predicted higher stiffness, yield force, and force at 20 mm, with a slight decrease in yield displacement. For the Ti alloy model, stiffness increased from 53.39 to 58.40 N/mm, yield force from 890.80 to 927.40 N, and the force at 20 mm from 933.23 to 986.73 N, while yield displacement decreased from 18.20 to 17.40 mm. For Ti Grade 23, stiffness rose from 54.56 to 59.48 N/mm, yield force from 894.00 to 930.00 N, and the force at 20 mm from 942.09 to 993.24 N, while yield displacement decreased from 17.90 to 17.15 mm.

Under 750 extension, experiment averaged stiffness 61.73 N/mm, yield displacement 18.93 mm, yield force 1,074.70 N, and force at 20 mm 1,112.73 N. In simulation, tightening the contact from frictionless → frictional → bonded increased stiffness and the force at 20 mm and slightly reduced yield displacement. Ti alloy: stiffness 62.60→69.32 N/mm, yield displacement 21.34→19.75 mm, yield force 1,241.17→1,263.73 N, force at 20 mm 1,193.10→1,273.50 N. Ti Grade 23: stiffness 64.03→70.73 N/mm, yield displacement 20.77→19.26 mm, yield force 1,232.37→1,255.33 N, force at 20 mm 1,205.30→1,283.60 N.

### 3.2 Assessment

#### 3.2.1 Equivalency of input parameters

The input parameters, including the material properties of the UHMWPE block and spinal rod, as well as temperature, loading speed, and the type and range of displacement control, were consistently maintained. However, in the case of the pedicle screw, although Titanium Grade 23 specified by the manufacturer was used, differences were observed between the outcomes of the comparator and the computational model.

#### 3.2.2 Output comparison

##### 3.2.2.1 Quantity

A total of four output variables were compared: stiffness, yield displacement, yield force, and force at 20 mm.

##### 3.2.2.2 Equivalency of output parameters

Types of outputs were equivalent.

##### 3.2.2.3 Rigor of output comparison

To ensure a rigorous evaluation, the comparison against the comparator incorporated the output uncertainty inherent in the computational model.

##### 3.2.2.4 Agreement of output comparison

A qualitative agreement was confirmed between the quantities of interest (QOIs) produced by the computational model and those derived from the comparator.

### 3.3 Applicability of the validation activities to the COU

#### 3.3.1 Relevance of the QOIs

The QOIs from the validation activities were identical to those for the COU.

#### 3.3.2 Relevance of the validation activities to the COU

The COU encompassed some of the validation points.

## 4 Discussion

### 4.1 Key findings

This study evaluated the mechanical behavior of spinal constructs under static compression (flexion) and static tension (extension) conditions using four different pedicle screw configurations. Experimental testing demonstrated consistent results across all loading modes, with minimal variability among the five specimens tested in each group, confirming the reliability of the experimental setup. In this study, the rigor of output comparison and agreement of output comparison was required to satisfy Level 3, corresponding to comparison by measuring the difference between computational results and experimental data, with differences being less than 10%.

Under tension conditions, some of the test and computational model results for yield displacement and yield force exceeded the 20 mm displacement threshold. These values were subsequently estimated through simulation to predict the expected mechanical behavior. Comparative analysis between experimental and computational results revealed consistent trends across both material types, titanium alloy and titanium grade 23. For both static compression and tension scenarios, the finite element models generally predicted higher stiffness and force values than the experimental results, with the discrepancies most pronounced under bonded contact conditions. Titanium grade 23 consistently exhibited slightly higher stiffness, yield force, and force at 20 mm than titanium alloy under identical contact conditions, reflecting its superior material properties.

### 4.2 Relation to the state of the art

Recent F1717-based computational studies frequently employ simplified or single-valued interface assumptions, which can mask the dominant role of contact in construct response (Chen et al., 2003; Xu et al., 2019; Chu et al., 2019; La Barbera et al., 2014). Our results extend this line of work by quantifying how interface modeling governs agreement, in line with contemporary credibility frameworks (Nagaraja et al., 2024; ASME V&V 40-2018, 2018; US Food and Drug Administration, 2023): across screws and loading modes, no single interface model satisfied all validation metrics simultaneously (Xu et al., 2019; La Barbera et al., 2014), and when construct stiffness and force at 20 mm are prioritized, a coefficient of friction of 0.10–0.20 produced the most consistent agreement with experiment, consistent with prevailing practice and tribological evidence for Ti-6Al-4V/UHMWPE pairs (Eastlack et al., 2023; Chen et al., 2003; Leksycki et al., 2022).

### 4.3 Quantitative agreement relative to a predefined target

We explicitly targeted ASME VandV 40 Level 3 comparison (model–experiment difference <10%). By screw type and loading mode, multiple contact settings (frictionless; μ = 0.1, 0.2, or 0.5; and bonded) met this criterion for stiffness and force at 20 mm, whereas bonded interfaces frequently exceeded it most notably for yield force. This pattern indicates that over-constrained interfaces inflate apparent construct strength and stiffness and should not be used as defaults when validating spinal construct models.

### 4.4 Contact effects and material trends

The influence of contact assumptions on the mechanical behavior was clearly demonstrated. As the coefficient of friction decreased from bonded to frictionless conditions, both stiffness and force at 20 mm displacement consistently decreased, while yield displacement increased. For the titanium alloy model, stiffness ranged from 47.13 to 62.60 N/mm under frictionless conditions and from 53.00 to 69.32 N/mm under bonded conditions. Similarly, for the titanium grade 23 model, stiffness ranged from 48.60 to 64.03 N/mm (frictionless) to 54.40–70.73 N/mm (bonded). Yield displacement exhibited an inverse trend relative to stiffness. For the titanium alloy model, yield displacement ranged from 18.20 to 22.53 mm under frictionless conditions and from 17.40 to 21.32 mm under bonded conditions. For titanium grade 23, yield displacement ranged from 17.90 to 23.04 mm (frictionless) to 17.15–21.30 mm (bonded).

In terms of yield force, the titanium alloy model produced values between 822.00 and 1,252.00 N under frictionless conditions and between 891.00 and 1,271.00 N under bonded conditions. Similarly, titanium grade 23 showed yield force values ranging from 853.00 to 1,243.33 N (frictionless) and from 903.40 to 1,274.67 N (bonded).

The force at 20 mm displacement followed the same trend as stiffness and yield force. For titanium alloy, force at 20 mm ranged from 848.58 to 1,193.10 N (frictionless) and from 933.66 to 1,273.50 N (bonded). Titanium grade 23 exhibited force at 20 mm values ranging from 877.07 to 1,205.30 N (frictionless) and from 950.36 to 1,283.60 N (bonded).

### 4.5 Per-screw validation summary

For the 640 screw under flexion, simulation results within 10% deviation from experimental data were observed across all contact conditions, namely, Frictionless, COF 0.1, COF 0.2, COF 0.5, and Bonded, for the titanium alloy. In the case of titanium grade 23, the criterion was satisfied under Frictionless, COF 0.1, COF 0.2, and COF 0.5 contact conditions. Under extension, only one case each was available for yield displacement and yield force, so these outputs were excluded from the comparison. Among the remaining metrics, the titanium alloy satisfied the 10% criterion under Frictionless, COF 0.1, COF 0.2, and COF 0.5 contact conditions. The titanium grade 23 met the threshold under Frictionless, COF 0.1, and COF 0.2.

For the 650 screw under flexion, no condition satisfied the 10% deviation criterion across all output variables. Although stiffness, yield displacement, and force at 20 mm showed acceptable values, the yield force exceeded 10% under all contact conditions. For extension, since yield displacement and yield force were not available, the comparison was based on the remaining outputs. In this case, the titanium alloy satisfied the 10% criterion under Frictionless, COF 0.1, COF 0.2, and COF 0.5, while titanium grade 23 met the threshold under Frictionless, COF 0.1, and COF 0.2.

For the 740 screw in flexion, all contact models for both materials were within 10% of experiment. In extension, the titanium alloy met the 10% threshold only with bonded contact, while titanium grade 23 met it with frictionless, COF 0.1, COF 0.2, and bonded contact.

For the 750 screw under flexion, titanium alloy frictionless contact conditions satisfied the 10% criterion. Although stiffness, yield displacement, and force at 20 mm showed acceptable values excluding the bonded condition, yield force exceeded the 10% threshold under all other conditions. For the extension mode, stiffness and force at 20 mm met the 10% criterion under Frictionless, COF 0.1, and COF 0.2 for titanium alloy, and under Frictionless, COF 0.1, and COF 0.2 for titanium grade 23. However, for yield displacement and yield force, the titanium alloy under the Frictionless condition showed deviations of 12.7% and 15.5%, respectively, and 4.3% and 17.6% under the Bonded condition. Titanium grade 23 exceeded 9.7% and 14.7% under the Frictionless condition, and 1.7% and 16.8% under the Bonded condition, respectively.

Collectively, these per-screw results indicate that no single contact model satisfied all output metrics across all screw types and loading modes; when construct stiffness and force at 20 mm displacement are adopted as the primary validation metrics, a coefficient of friction of 0.10–0.20 is recommended.

### 4.6 Uncertainty and robustness

Because screw geometries were reverse-engineered from commercial implants, exact material parameters (e.g., E, σy, hardening) and screw–block contact properties (friction law and μ) represent epistemic uncertainties. Treating these as uncertain inputs clarifies why some cases align with Grade 23 properties while others are better matched by a lower effective modulus. We therefore recommend explicit reporting and justification of contact/material assumptions and, where feasible, experimental calibration of μ and material parameters to strengthen reproducibility.

### 4.7 Practical implication

For F1717 simulations intended for decision-making or regulatory submissions, we recommend avoiding fully bonded interfaces as a default, documenting the friction law and μ, and selecting values preferably μ = 0.10–0.20 that are justified by experiment for the specific construct. Overall, the computational models qualitatively captured experimental trends, but tended to overestimate force- and stiffness-related outputs under bonded assumptions, whereas displacement-related outputs showed moderate sensitivity. Overly simplified interfaces may thus lead to significant overestimations; future work should incorporate experimentally validated friction models and refined interface characterizations to enhance simulation fidelity.

## 5 Limitations

This study followed the ASTM F1717-15 standard configuration, which uses UHMWPE surrogate blocks and intentionally idealizes the bone implant environment. As such, the accuracy reported here should not be over generalized to vertebral anatomy, bone heterogeneity, or fusion interfaces. Our tests and simulations were limited to static compression (flexion) and static tension (extension); dynamic loading and fatigue behavior were not assessed, so durability under cyclic conditions remains outside the present scope.

The pedicle screw geometries were reconstructed from commercial implants, so the exact material properties (for example, Young’s modulus, yield strength, and hardening behavior) were not directly measured and should be regarded as epistemic uncertainties. In addition, the screw-block interface was not calibrated through independent tribological testing. Instead, we explored literature based friction coefficients (0.1–0.5) together with bounding assumptions (bonded and frictionless) to examine contact sensitivity, which leaves residual uncertainty in the interface model.

Future work will broaden validation to cadaveric or bone mimicking constructs, incorporate the F1717 fatigue protocol and other dynamic loading conditions, quantify the effects of set screw preload and torque, and expand the range of materials and surface conditions (for example, CoCr, coatings, roughness). We also plan to experimentally calibrate and identify key material and contact parameters to reduce uncertainty and improve generalizability.

## 6 Conclusion

This study validated finite element models of pedicle screw constructs under static flexion and extension in accordance with ASTM F1717-15, with verification and uncertainty quantification incorporated to support model credibility. Experimental repeatability was high (maximum deviations: stiffness 6.4%, yield displacement 9.1%, yield force 7.2%, force at 20 mm 7.5%).

Across configurations, the models reproduced the observed trends but tended to over-predict stiffness- and load-related outputs when fully bonded interfaces were assumed. The largest deviations reached 19.8% for stiffness, 21.5% for yield force, and 18.4% for force at 20 mm; yield-displacement errors peaked at 14.2% under frictionless assumptions. These patterns underscore the central role of interface definition: force and stiffness were highly sensitive to contact conditions, whereas displacement metrics showed moderate variability.

When construct stiffness and force at 20 mm are adopted as primary validation targets, a coefficient of friction in the range 0.10–0.20 provided the most consistent agreement with experiment across screws and loading modes. For decision-making or regulatory use, we recommend avoiding fully bonded interfaces as a default, explicitly reporting and justifying the chosen friction law and coefficient, and, where feasible, experimentally calibrating key material and contact parameters. These steps should improve predictive reliability and facilitate broader adoption of F1717-based simulations in practice.

## Data Availability

The original contributions presented in the study are included in the article/supplementary material, further inquiries can be directed to the corresponding author.
